# The potential role of methyltransferase-like 5 in deficient mismatch repair of uterine corpus endometrial carcinoma

**DOI:** 10.1080/21655979.2022.2036912

**Published:** 2022-02-15

**Authors:** Xiaojuan Liu, Hui Ma, Lisha Ma, Kun Li, Yanhua Kang

**Affiliations:** a Department of Gynaecology and Obstetrics,The First Affiliated Hospital of Hebei North University, Zhangjiakou, Hebei Province, China; b Department of Quality Control Office, Zhangjiakou Infectious Disease Hospital, China

**Keywords:** Uterine corpus endometrial carcinoma, mismatch repair, methyltransferase like 5, pathology, cancer

## Abstract

To explore the potential function of methyltransferase-like 5 (METTL5) in uterine corpus endometrial carcinoma (UCEC) and verify the relationship between deficient DNA mismatch repair (MMR) and METTL5. We used bioinformatics to predict the possible role of METTL5 and molecular biology methods to analyze METTL5 expression. We observed UCEC proliferation, development, and apoptosis using a METTL5 knockdown lentivirus and, coupled with METTL5 bioinformatics and Western blot analysis, detected microsatellite instability (MSI) and MMR. Gene ontology (GO) and Kyoto Encyclopedia of Genes and Genomes (KEGG) analyses were performed. Finally, some METTL5-associated gene mutations in UCECs were detected. Results show that METTL5 expression in UCEC tumor tissue was increased, and UCEC patients with high METTL5 expression had worse prognostic outcomes. We also observed the highest METTL5 expression level in KLE cells. Furthermore, knocking down METTL5 weakened the proliferation, reduced tumor volume and biomarkers, and increased apoptosis. Moreover, METTL5 knockdown induced the MSH2, MSH6 and PMS2 expression in MMR. METTL5 was negatively correlated with gene silencing, mRNA binding, olfactory receptor activity, antigen processing and presentation, cytosolic DNA sensing, olfactory transduction, and RIG-1-like and Toll-like receptor signaling pathways. METTL5 may regulate MMR protein levels in UCECs, thus enhancing UCEC proliferation, development, and prognosis.

## Graphical Abstract

**Figure:**
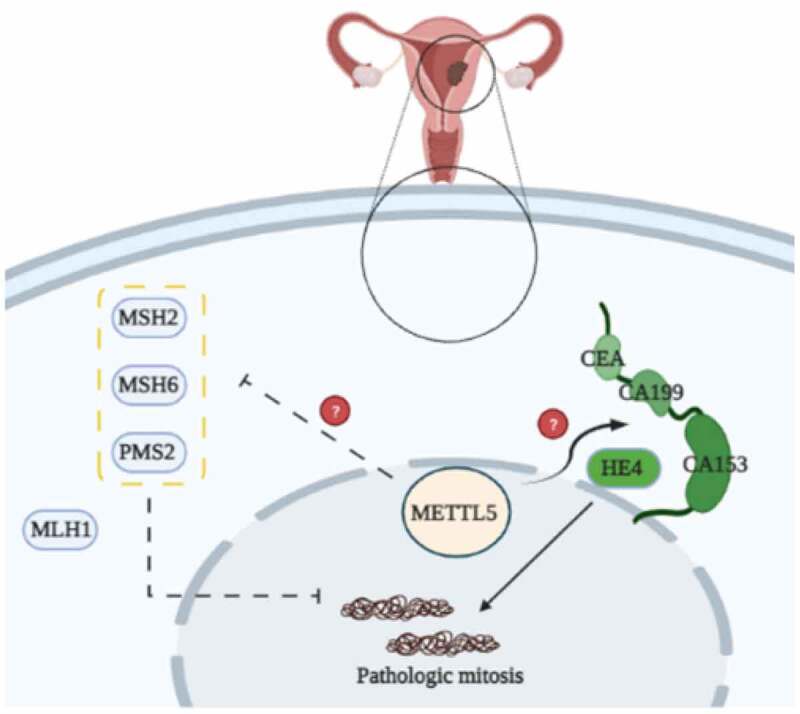

## Introduction

Uterine corpus endometrial carcinoma (UCEC) is the most common and threatening gynecological malignant tumor that leads to death due to its high recurrence rate [1]. Endometrioid carcinoma (EC) is characterized by a proliferative endometrium, cellular pleomorphism, and glandular complexity [2]. For patients with EC of intermediate or high recurrence risk, radical hysterectomy combined with pelvic and para-aortic lymphadenectomy is recommended [3]. Evolving medical technology has slowed the long-term mortality declines associated with EC. However, EC mechanisms and treatments still need continuous exploration.

Deficient DNA mismatch repair (MMR), a strong mutator phenotype of microsatellite instability (MSI), and has recently been described in ECs [4,5]. In addition, biological relevance, modal classification, and genomic instability complexities underlying EC tumorigenesis are reportedly related to MSI and MMR [6].

As previously reported, regulating MMR may be a prognostic factor for tumor recurrence and adjuvant chemoradiotherapy in patients with advanced UCECs [7,8]. Therefore, it is necessary to identify MMR targets for UCEC treatment and prevention.

Methyltransferase-like 5 (METTL5) is a protein-coding gene associated with intellectual developmental disorder, autosomal recessive 72 and primary autosomal recessive microcephaly. It is related to nucleic acid binding and protein methyltransferase activity [9–11]. Recent reports suggest that METTL5 can regulate breast cancer and lung adenocarcinoma translation initiation and immune implications [12,13]. However, the role of METTL5 in UCEC MMR is not yet fully understood.

We aimed to investigate the potential role of METTL5 in UCECs, then used a METTL5 knockdown lentivirus to regulate MMR and UCEC development in vivo and in vitro. The present findings indicate that METTL5 may regulate MMR in UCECs. These results may provide novel strategies for UCEC treatment via MMR-associated mechanisms.

## Materials and methods

### Human tumor specimens

We selected 16 patients from the Department of Pathology in our hospital and pathologically diagnosed them with UCEC from May 2019 to May 2021. We obtained adjacent para-carcinoma tissues as controls. All patients provided informed consent, and the Ethics Committee of The First Affiliated Hospital of Hebei North University approved the experimental design.

### Bioinformatics analysis

We determined METTL5 expression using the TIMER2.0 website (http://timer.cistrome.org/). The survival assay of the UCEC patients in the METTL5 low and METTL5 high expression groups was performed using the Kaplan-Meier plotter website (http://kmplot.com/analysis/), and the cutoff of expression was 50%. We downloaded the Pan-Cancer Assay from The Cancer Genome Atlas (TCGA) database, as described previously [14], and used a target gene assay to analyze genes related to mutation and prognosis via the website https://www.mutarget.com/analysis?type=target.

### Cell lines and antibodies

This study used normal endometrial cells (NECs), KLE cells, RL952 cells, Ishikawa cells, and ECC-1 cells. They were maintained and cultured as previously described [15]. The cell lines used in this study were as follows: normal endometrial cells (NEC, CP-H058, Procell, Wuhan, China), KLE (CL-0133, Procell, Wuhan, China), RL952 (CL-0197, Procell, Wuhan, China), Ishikawa (CL-0283, Procell, Wuhan, China), and ECC-1 (BS-C163325, BinSuiBio, Shanghai, China).

The following antibodies were used: Anti-METTL5 (16791-1-AP, Proteintech, Rosemont, USA), anti-Ki-67 (ab15580, Abcam, Cambridge, USA), anti-CEA (ab207718, Abcam, Cambridge, USA), anti-CA199 (ab3982, Abcam, Cambridge, USA), anti-CA153 (ab109185, Abcam, Cambridge, USA), anti-HE4 (13, ab200828, Abcam, Cambridge, USA), anti-MLH1 (ab92312, Abcam, Cambridge, USA), anti-MSH2 (ab212188, Abcam, Cambridge, USA), anti-MSH6 (ab92471, Abcam, Cambridge, USA), anti-PMS2 (ab110638, Abcam, Cambridge, USA), and anti-β-actin (M01263-2, Boster, Wuhan, China).

### Animals

Chongqing Tengxin Biotechnology Co., Ltd. (Chongqing, China) supplied four-week-old healthy male Balb/c mice. We subjected the mice to specific pathogen-free (SPF) conditions and housed them under the Guide for the Care and Use of Laboratory Animals. The Ethics Committee of the First Affiliated Hospital of Hebei North University approved the study design (Protocol No.: 2020ECHNU033).

### METTL5 knockdown lentivirus administration

We designed and chemically synthesized METTL5 knockdown lentiviruses (sh-METTL5) and control lentiviruses (GenePharma Corporation, Shanghai, China) and stored them at −80°C. Lentivirus cell administration has been well-established [16]. Table 1 lists the METTL5 lentivirus sequences.

**Table 1.** The METTL5 lentivirus sequences

Name	Sequence
sh-METTL5-182	5’-gcttaaggaactagagagtc-3’
sh-METTL5-361	5’-ttagcatcggaactgcaatg-3’
sh-METTL5-729	5’-catcatacaagtttcacaaa-3’
Negative Control	5’-ccccgcaaacaaaagtcgtt-3’
Control-shMETTL5	5’-gcttccggcaccggccgagg-3’

The subjects were randomly divided into four groups (n = 6 in each group) [1]: Con group: untreated KLE cells or mice injected with KLE cells [2]; sh-METTL5 group: KLE sh-METTL5 lentivirus cells or mice injected with KLE sh-METTL5 lentivirus cells [3]; Control-shMETTL5 group: lentivirus cells with METTL5 control lentivirus or mice injected with lentivirus cells with METTL5 control lentivirus.

### UCEC mouse model

The mice were subcutaneously injected with KLE cells with or without sh-METTL5 lentivirus administration. After five days, we observed their vital signs and inoculation sites, as described previously [17]. The mice were sacrificed by humanization strategy, including rapid weight loss (> 20%), metastatic burden, hunching, dehydration, and labored breathing.

### Western blotting

A total of 50 μg of protein was transferred to nitrocellulose membranes, as described previously [18]. These were developed by the Bio-Rad imaging system and quantized using Image Lab software (version 3.0).

### Immunohistochemistry

Sections were incubated with METTL5 and Ki-67 primary antibodies at 4°C overnight, as previously described [19]. Immunohistochemical analysis was mainly based on visual observation of the number and intensity of staining positive cells.

### Propidium iodide (PI)

Cells from each group were analyzed using a PI-Hoechst assay (40755ES64, Qcbio Science&Technologies Co., Ltd, Shanghai, China), as described previously [20].

### Terminal deoxynucleotidyl transferase dUTP nick end labeling (TUNEL) staining

Tissue fractions from each group were analyzed using a TUNEL assay (ab66108, Abcam, Cambridge, UK), as described previously [21].

### 3-(4,5)-dimethylthiahiazo (-z-y1)-3,5-di- phenytetrazoliumromide (MTT)

Cell viability was studied using the MTT assay, and absorbance was measured at 490 nm, as described previously [22].

### Statistical analysis

GraphPad Prism 6.0 and SPSS 18.0 were used to analyze the data, which are expressed as the mean ± standard deviation (SD). One-way or two-way ANOVAs were performed and multiple comparisons are analyzed using a Holm-Sidak test. A P-value < 0.05 was considered statistically significant.

## Results

To explore the potential function of METTL5 UCEC and verify the relationship between MMR and METTL5. We used bioinformatics to predict the possible role of METTL5 and molecular biology methods to analyze METTL5 expression. We observed that the proliferation, development, and apoptosis using a METTL5 knockdown lentivirus and coupled with METTL5 bioinformatics and Western blot analysis, detected MSI and MMR.

### METTL5 was over-expressed and increased the risk of UCEC survival

**Figure 1.**
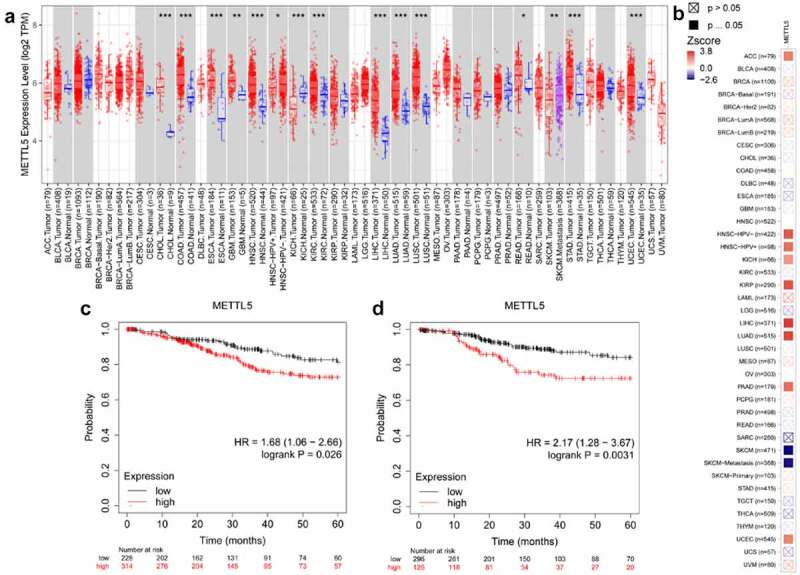
Bioinformatics analysis of METTL5 in UCECs. (a) METTL5 expression in different tissues. (b) METTL5 risk assay. (c) OS analyses, and (d) RFS analyses of high and low METTL5 levels from UCEC patients.

Figure 1 shows the predicted role of METTL5 in UCECs detected by TIMER2.0 and the Kaplan-Meier plotter system. Figure 1(a) shows that TIMER2.0 quantified METTL5 levels in different types of cancers, and METTL5 levels in UCEC tumor tissues were increased (P < 0.05). TIMER2.0 also showed that METTL5 increased the risk of UCEC survival (Figure 1(b)). For overall survival (OS) and recurrence-free survival (RFS) analyses, we could easily consider that, given the high METTL5 level state (Figure 1(c,d)), UCEC patients would have a worse prognostic outcome (P < 0.05).

### METTL5 levels were different in UCEC tumor tissues, para-tumor tissues, and cell lines

**Figure 2.**
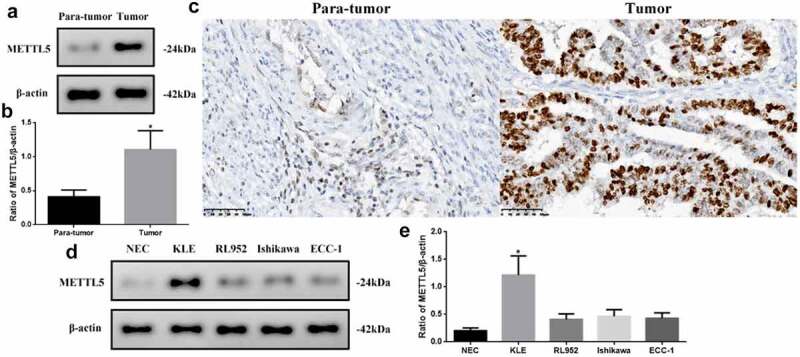
METTL5 levels in different UCEC tissues or cell lines. (a) METTL5 expression in para-tumor and tumor tissues by Western blot. (b) Western blot quantification, and (c) IHC for METTL5 (×400). (d) METTL5 expression in NEC, KLE, RL952, Ishikawa, and ECC-1 cells. (e) METTL5 quantification. Protein levels were normalized to β-actin. (Tumor vs. Para-tumor, KLE vs. other cells, *P < 0.05, n = 6 per group).

Bioinformatic analysis results suggest that METTL5 may be a gene related to UCECs, as we detected varying METTL5 levels in different tissues and cells (Figure 2). METTL5 levels in tumors were significantly higher than in para-tumor tissues (P < 0.05, Figure 2(a,b)). The IHC designed outcomes (Figure 2(c)) approximately localized METTL5 expression to the nucleus. METTL5 levels in KLE cells were the highest (P < 0.05) compared to the others (Figure 2(d,e)). Therefore, we chose KLE for follow-up METTL5-associated experiments.

### Knocking down METTL5 regulated UCEC development in vitro

**Figure 3.**
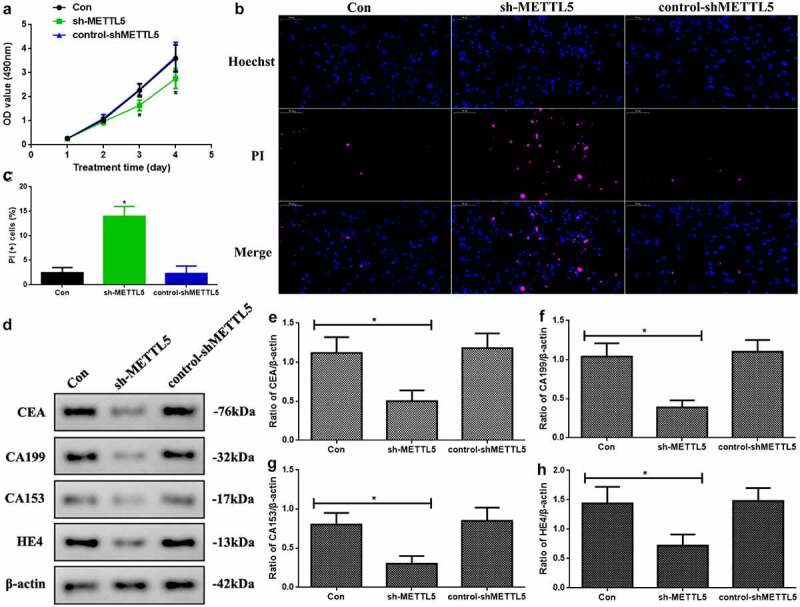
Knocking down METTL5 regulated UCEC development in vitro. (a) MTT over four days in different groups. (b) PI-Hoechst (×400) and (c) Quantification of PI (+) cells. (d) CEA, CA199, CA153, and HE4 expression. (e-h) CEA, CA199, CA153, and HE4 expression quantification. (sh-METTL5 vs. other groups, *P < 0.05, n = 6 per group).

As the previous results indicated that METTL5 might be correlated with UCEC progression, we designed a method to block METTL5 levels: sh-METTL5 (Figure 3). We observed proliferation in the different groups (Figure 3(a)). Over three to four days, the absorbance OD value decreased in the sh-METTL5 group (P < 0.05). Furthermore, PI-Hoechst was used to observe the apoptotic function of METTL5 (Figure 3(b)), and the apoptotic level in the sh-METTL5 group was increased (Figure 3(c), p < 0.05). Lastly, CEA, CA199, CA153, and HE4 levels, which are UCEC tumor biomarker levels, were reduced in the sh-METTL5 group (P < 0.05, Figure 3(d-h)).

### Knocking down METTL5 regulated UCEC development in vivo

**Figure 4.**
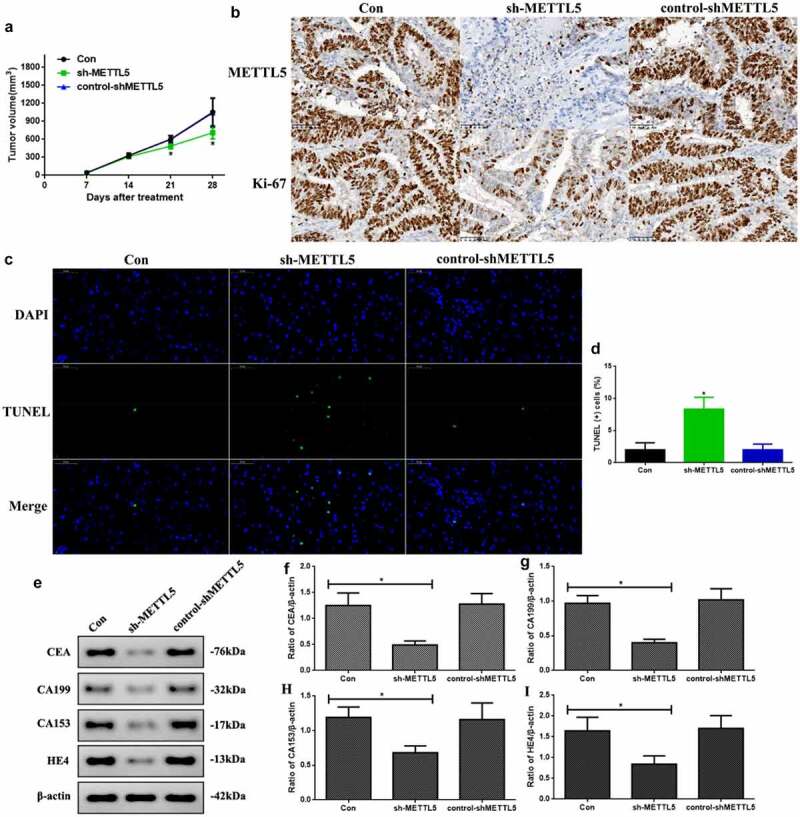
Knocking down METTL5 regulated UCEC development in vivo. (a) Tumor volume over a 28 day period. (b) IHC for METTL5 and Ki-67. (c) TUNEL-DAPI (×400) and (d) Quantification of TUNEL (+) cells. (e) CEA, CA199, CA153, and HE4 expression. (f-i) CEA, CA199, CA153, and HE4 expression quantification. (sh-METTL5 vs. other groups, *P < 0.05, n = 6 per group).

As knocking down METTL5 administration regulated UCEC development, we repeated the process in vivo. First, the tumor volumes at 21 and 28 days were significantly decreased in sh-METTL5 mice (P < 0.05, Figure 4(a)). Figure 4(b) shows METTL5 expression and the proliferation index. Secondly, METTL5 and Ki-67 expression were reduced by lentivirus knockdown administration. Third, apoptotic levels in sh-METTL5 mice were increased in the TUNEL assay (Figure 4(c,d), P < 0.05). Lastly, CEA, CA199, CA153, and HE4 expression in sh-METTL5 mice was reduced (P < 0.05, Figure 4(e-i)).

### METTL5 is related to MSI and MMR in UCECs according to Pan-Cancer and correlation analyses

**Figure 5.**
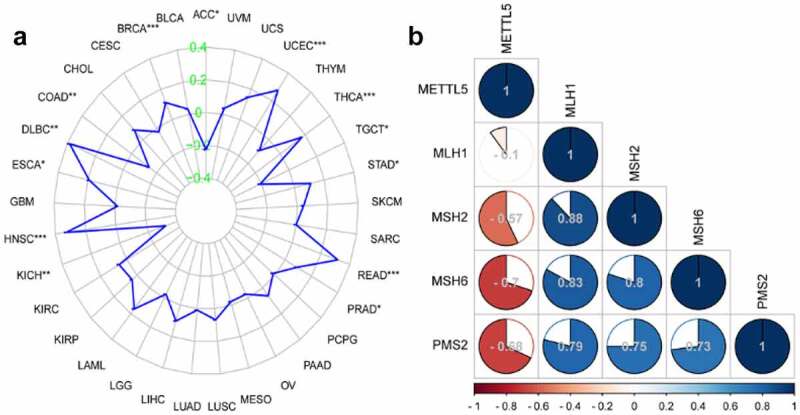
Bioinformatics analysis of METTL5 in MSI and MMR in UCECs by Pan-Cancer and correlation analyses. (a) Pan-Cancer analysis of MSI assay in different cancers. (b) Correlation assay for METTL5 with MLH1, MSH2, MSH6, and PMS2 in UCECs.

We used Pan-Cancer and correlation analyses to detect the role of METTL5 in MSI and MMR in UCECs. Figure 5(a) shows the function of METTL5 in MSI in different cancers. We confidently concluded that METTL5 was positively related to MSI in UCECs. Correlation analysis verified that METTL5 was negative for the MMR markers MSH2, MSH6, and PMS2 but positive for MLH1 in UCECs (Figure 5(b)).

### Knocking down METTL5 increased MMR marker levels in vivo

**Figure 6.**
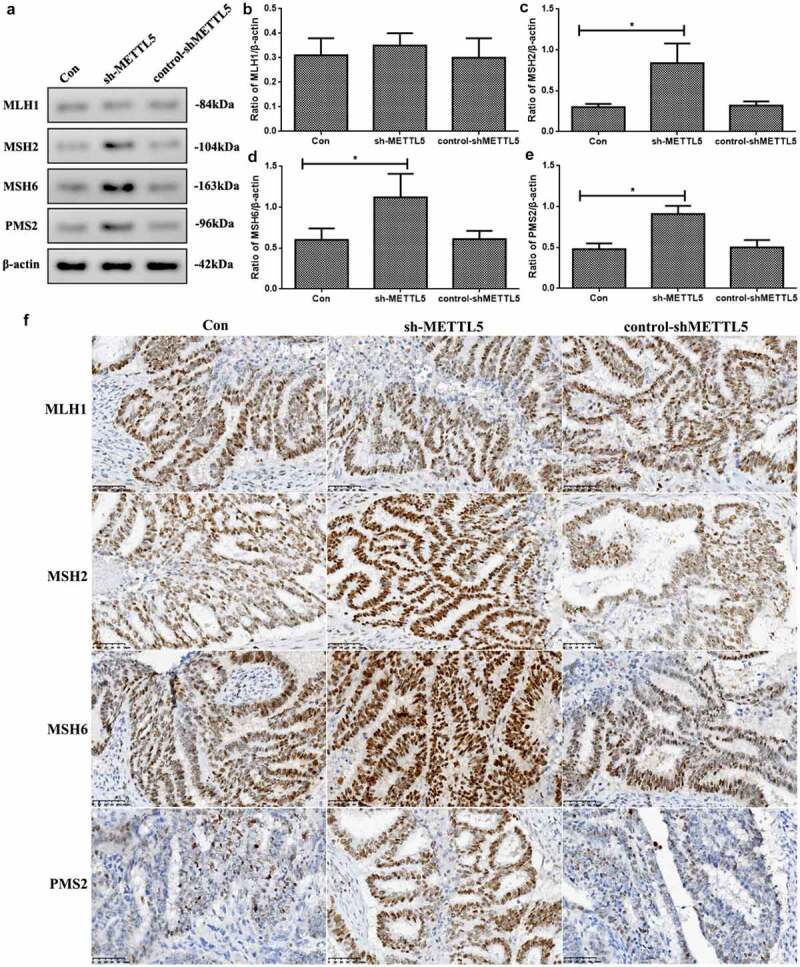
Knocking down METTL5 increased the markers’ MMR level in vivo. (a) MLH1, MSH2, MSH6, and PMS2 expression in vivo. (b-e) MLH1, MSH2, MSH6, and PMS2 expression quantification. (f) IHC assay of MLH1, MSH2, MSH6, and PMS2 expression. (sh-METTL5 vs. other groups, *P < 0.05, n = 6 per group).

As METTL5 was predicted to be a gene related to MSI and MMR in UCECs based on previous results, we detected MMR marker levels in vivo. Figure 6(a) shows induced MSH2, MSH6, and PMS2 expression in the sh-METTL5 group (P < 0.05, Figure 6(b,e)). Figure 6(f) shows the IHC outcomes (Figure 6(c)).

### Greater potential role of METTL5 in Gene Ontology (GO) and Kyoto Encyclopedia of Genes and Genomes (KEGG) analyses by Pan-Cancer

**Figure 7.**
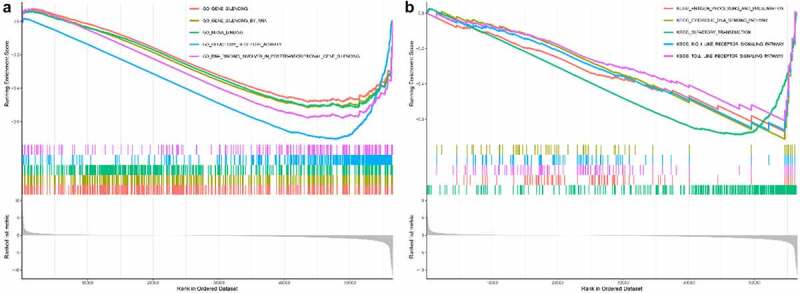
Bioinformatics analysis of METTL5 in GO and KEGG by Pan-Cancer. (a) GO assay of METTL5 by Pan-Cancer assay. (b) METTL5 KEGG assay.

In the GO assay, we found that METTL5 was negatively correlated with gene silencing, gene silencing by RNA, mRNA binding, olfactory receptor activity, and RNA binding involved in post-transcriptional gene silencing (Figure 7(a)). In the KEGG assay, METTL5 was negatively correlated with antigen processing and presentation, cytosolic DNA sensing pathways, olfactory transduction, and RIG-1-like and Toll-like receptor signaling pathways (Figure 7(b)).

### Further METTL5 target genes in UCEC patients

**Figure 8.**
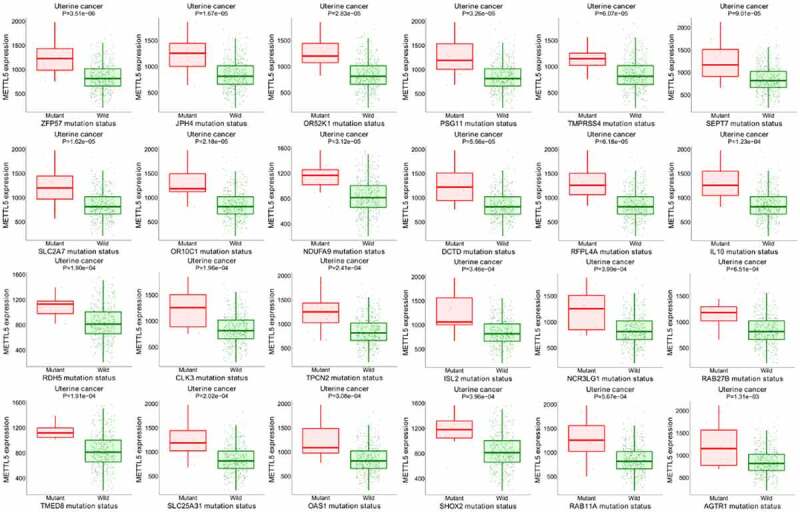
Further bioinformatics analysis of METTL5 in UCEC patients by target gene system. (a) METTL5 expression in AZFP59, JPH4, OR52K1, PSG11, TMPRSS4, SEPT7, SLC2A7, OR10C1, NDUFA9, DCTD, RFPL4A, IL10, RDH5, CLK3, TPCN2, ISL2, NCR3LG1, RAB27B, TMED8, SLC25A31, OAS1, SHOX2, RAB11A, and AGTR1 mutant or wild-type UCEC patients.

Figure 8 shows the METTL5 target gene assay in UCECs, where pink represents mutant patients and green represents wild patients. In the UCEC patients with higher METTL5 levels, ZFP59, JPH4, OR52K1, PSG11, TMPRSS4, SEPT7, SLC2A7, OR10C1, NDUFA9, DCTD, RFPL4A, IL10, RDH5, CLK3, TPCN2, ISL2, NCR3LG1, RAB27B, TMED8, SLC25A31, OAS1, SHOX2, RAB11A, and AGTR1 mutations were found. These results suggest that METTL5 is related to these gene mutations and is involved in UCEC prognosis.

**Figure 9.**
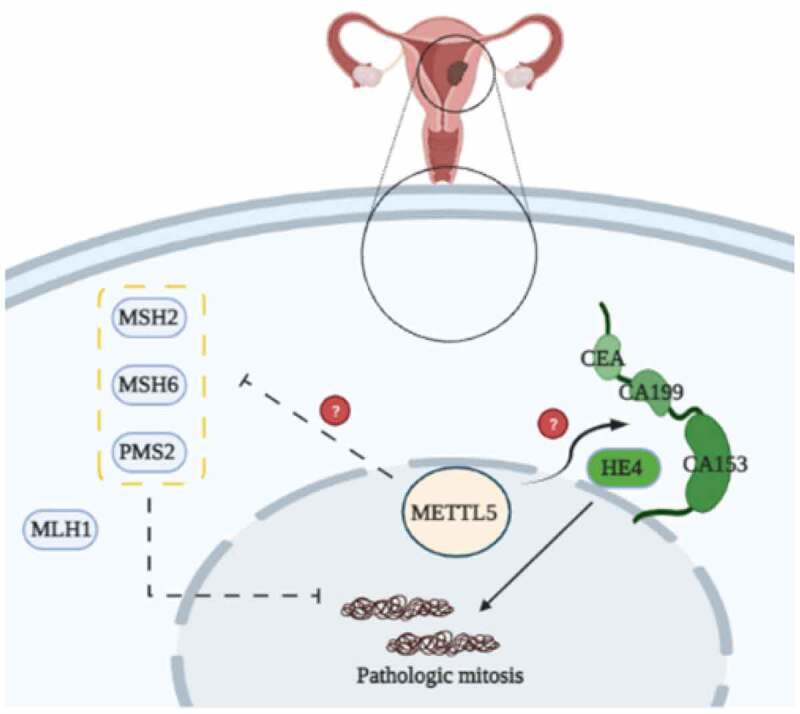
The potential mechanisms of METTL5 by MMR proteins in UCECs. METTL5-mediated MMR regulation in UCECs. METTL5 may regulate MSH2, MSH6, and PMS2 expression, weaken MMR and MSI progress, and increase pathological mitosis in UCEC cells.

Figure 9 shows the potential mechanisms of METTL5 mediated by MMR proteins in UCECs. METTL5 likely regulates MMR proteins, such as MSH2, MSH6, and PMS, enhances UCEC proliferation and development, and is involved in prognosis.

## Discussion

EC is a threatening gynecological malignant tumor associated with a 20% mortality rate in Western and developing countries [2,23,24]. EC mortality is still increasing and needs more effective diagnostic and treatment strategies [25].

In short repetitive DNA sequences, MSI refers to the hypermutator phenotype second to frequent polymorphisms and single nucleotide substitutions. It is a consequence of DNA MMR deficiency and vital to ECs [5,26].

Liu J, et al investigated a hotspot R298P mutation in UCEC was present in a key component of METTL14, exhibiting reductions in m6A methylation that are likely due to either this METTL14 mutation or reduced expression of METTL3 [27]. Qin L, et al discovered Wilms’ tumor 1-associating protein knocking down could up-regulate level of caveolin-1 and METTL3 at 3’-untranslated region in EC cells [28]. These studies shown Methyltransferase-like family may be a strong association with endometrial cancer. METTL5 is a catalytic subunit of a heterodimer with TRMT112, which specifically methylates the sixth adenine at position 1832 of 18S rRNA [9]. Previous reports associate METTL5 with an intellectual developmental disorder, primary autosomal recessive microcephaly, and protein methyltransferase activity [9–11]. Recent studies report that METTL5 could be related to cancer [12,13]. Insofar, there is very little research on METTL5, but it will be relevant in oncology research. In our study, we used bioinformatics analysis and molecular biology methods to explore the function of METTL5 in UCECs. METTL5 expression in UCEC tumor tissue increased, and with high METTL5 isoform expression, patients with UCECs had poor prognostic outcomes. These results show that METTL5 may be an oncogene involved in UCEC prognosis. To verify this, we observed a difference in METTL5 protein levels. METTL5 expression levels increased in ECs and KLE cells, consistent with the bioinformatics analysis. Interestingly, METTL5 is expressed in the nucleus. Yanli Z, et al found that METTL13 was significantly abnormally expressed in endometrial cancer tissue samples [29]. Moreover, Zhilin Z, et al considered METTL16 had a low hazard ratio in UCEC by Multivariate Cox regression analysis [30]. Different from METTL13 and METTL16, METTL5 may be a carcinogenic factor and involved in UCEC prognosis which was first reported,

Apoptosis is a programmed cell death process without any inflammatory responses [31]. We knocked down METTL5 levels using a lentivirus to explore the role of METTL5 in apoptosis and tumor progression in EC. In our study, knocking down METTL5 weakened proliferation, reduced tumor volume and biomarker levels, and increased apoptosis. These results demonstrated that METTL5 could regulate apoptosis and tumor progression in UCECs and are consistent with the bioinformatic analysis outcomes above.

Through Pan-Cancer and correlation analyses, we found that METTL5 was positively correlated with MSI and negatively correlated with MRR proteins, especially MSH2, MSH6, and PMS2. There is no research on METTL5 with MRR proteins. Hence, we have only explored this process according to our bioinformatics results. We used Western blotting and IHC to prove our bioinformatic outcomes related METTL5 to MMR. Our results showed that sh-METTL5 could improve MSH2, MSH6, and PMS2 expression but not MLH1 expression.

First, we will report the results. However, some methodological deficiencies due to our limited lab conditions must be taken into consideration. Furthermore, more rigorous and advanced experiments by other scholars are required.

In further bioinformatics analysis of METTL5 in UCEC patients using Pan-Cancer and the target gene system, we found that METTL5 was negatively correlated with gene silencing, mRNA binding, olfactory receptor activity, antigen processing and presentation, cytosolic DNA sensing in GO, antigen processing and presentation, cytosolic DNA sensing pathways, olfactory transduction, and RIG-1-like and Toll-like receptor signaling pathways in KEGG.

Finally, METTL5 may be involved in UCEC prognosis and related to ZFP59, JPH4, OR52K1, PSG11, TMPRSS4, SEPT7, SLC2A7, OR10C1, NDUFA9, DCTD, RFPL4A, IL10, RDH5, CLK3, TPCN2, ISL2, NCR3LG1, RAB27B, TMED8, SLC25A31, OAS1, SHOX2, RAB11A, and AGTR1 mutations. Several studies are needed to verify this data, which will be the focus of our subsequent experiments.

A limitation of this study is that METTL5 mRNA levels should be detected in endometrial cells to explain whether differences in METTL5 expression are due to transcription or translation levels.

## Conclusion

Our study found that knocking down METTL5 could significantly activate apoptosis and inhibit EC development via MMR administration. Hypothetically, our findings may offer a new approach for UCEC treatment by regulating MMR.

## References

[1] Wang P, Zeng Z, Shen X, et al. Identification of a Multi-RNA-Type-Based Signature for Recurrence-Free Survival Prediction in Patients with Uterine Corpus Endometrial Carcinoma. DNA Cell Biol. 2020;39(4):615–630. 32105510 10.1089/dna.2019.5148

[2] Akhtar M, Al Hyassat S, Elaiwy O, et al. Classification of Endometrial Carcinoma: new Perspectives Beyond Morphology. Adv Anat Pathol. 2019;26(6):421–427. 31567131 10.1097/PAP.0000000000000251

[3] Todo Y, Kato H, Kaneuchi M, et al. Survival effect of para-aortic lymphadenectomy in endometrial cancer (SEPAL study): a retrospective cohort analysis. Lancet. 2010;375(9721):1165–1172. 20188410 10.1016/S0140-6736(09)62002-X

[4] Stelloo E, Nout RA, Osse EM, et al. Improved Risk Assessment by Integrating Molecular and Clinicopathological Factors in Early-stage Endometrial Cancer-Combined Analysis of the PORTEC Cohorts. Clin Cancer Res. 2016;22(16):4215–4224. 27006490 10.1158/1078-0432.CCR-15-2878

[5] Leon-Castillo A, Britton H, McConechy MK, et al. Interpretation of somatic POLE mutations in endometrial carcinoma. J Pathol. 2020;250(3):323–335. 31829442 10.1002/path.5372 PMC7065171

[6] Eto T, Zhao Y, Maruyama A, et al. Modal variety of microsatellite instability in human endometrial carcinomas. J Cancer Res Clin Oncol. 2016;142(2):353–363. 26298837 10.1007/s00432-015-2030-2 PMC4717170

[7] Jones NL, Xiu J, Rocconi RP, et al. Immune checkpoint expression, microsatellite instability, and mutational burden: identifying immune biomarker phenotypes in uterine cancer. Gynecol Oncol. 2020;156(2):393–399. 31882243 10.1016/j.ygyno.2019.11.035

[8] Willvonseder B, Stogbauer F, Steiger K, et al. The immunologic tumor microenvironment in endometrioid endometrial cancer in the morphomolecular context: mutual correlations and prognostic impact depending on molecular alterations. Cancer Immunol Immunother. 2021;70(6):1679–1689. 33340331 10.1007/s00262-020-02813-3 PMC8139910

[9] van Tran N, Ernst FGM, Hawley BR, et al. The human 18S rRNA m6A methyltransferase METTL5 is stabilized by TRMT112. Nucleic Acids Res. 2019;47(15):7719–7733. 31328227 10.1093/nar/gkz619 PMC6735865

[10] Ignatova VV, Stolz P, Kaiser S, et al. The rRNA m(6)A methyltransferase METTL5 is involved in pluripotency and developmental programs. Genes Dev. 2020;34(9–10):715–729. 32217665 10.1101/gad.333369.119 PMC7197354

[11] Richard EM, Polla DL, Assir MZ, et al. Bi-allelic Variants in METTL5 Cause Autosomal-Recessive Intellectual Disability and Microcephaly. Am J Hum Genet. 2019;105(4):869–878. 31564433 10.1016/j.ajhg.2019.09.007 PMC6817559

[12] Rong B, Zhang Q, Wan J, et al. Ribosome 18S m(6)A Methyltransferase METTL5 Promotes Translation Initiation and Breast Cancer Cell Growth. Cell Rep. 2020;33(12):108544. 33357433 10.1016/j.celrep.2020.108544

[13] Sun S, Fei K, Zhang G, et al. Construction and Comprehensive Analyses of a METTL5-Associated Prognostic Signature With Immune Implication in Lung Adenocarcinomas. Front Genet. 2020;11:617174. 33679869 10.3389/fgene.2020.617174 PMC7933593

[14] Stadler ZK, Maio A, and Chakravarty D, et al. Therapeutic Implications of Germline Testing in Patients With Advanced Cancers. J Clin Oncol. 2021;39(24):2698-2709. 10.1200/JCO.20.03661 PMC8376329 34133209

[15] Fernando SR, Lee CL, Wong BP, et al. Expression of membrane protein disulphide isomerase A1 (PDIA1) disrupt a reducing microenvironment in endometrial epithelium for embryo implantation. Exp Cell Res. 2021;405(2):112665. 34111473 10.1016/j.yexcr.2021.112665

[16] Li GZ, Deng JF, Qi YZ, et al. COLEC12 regulates apoptosis of osteosarcoma through Toll-like receptor 4-activated inflammation. J Clin Lab Anal. 2020;34(11):e23469. 32822099 10.1002/jcla.23469 PMC7676208

[17] Liu D, Gunther K, Enriquez LA, et al. ROR1 is upregulated in endometrial cancer and represents a novel therapeutic target. Sci Rep. 2020;10(1):13906. 32807831 10.1038/s41598-020-70924-z PMC7431863

[18] Ma M, Chai K, Deng R. Study of the correlation between the noncanonical pathway of pyroptosis and idiopathic inflammatory myopathy. Int Immunopharmacol. 2021;98:107810. 34116285 10.1016/j.intimp.2021.107810

[19] Jiang L, Niu W, Zheng Q, et al. Identification of an Autoantibody Against ErbB-3-Binding Protein-1 in the Sera of Patients With Chronic Hepatitis B Virus Infection. Front Immunol. 2021;12:640335. 34113340 10.3389/fimmu.2021.640335 PMC8185336

[20] Chu Q, Wang S, Jiang L, et al. Patulin induces pyroptosis through the autophagic-inflammasomal pathway in liver. Food Chem Toxicol. 2021;147:111867. 33217525 10.1016/j.fct.2020.111867

[21] Liu Q, Chen L, Liang X, et al. Exercise attenuates angiotensin-induced muscle atrophy by targeting PPARgamma/miR-29b. J Sport Health Sci. 2021; DOI:10.1016/j.jshs.2021.06.002. PMC9729927 34116237

[22] Wang Z, Xu J, Wang Y, et al. Total saponins from Tupistra chinensis baker inhibits growth of human gastric cancer cells in vitro and in vivo. J Ethnopharmacol. 2021;278:114323. 34116191 10.1016/j.jep.2021.114323

[23] Hussein YR, Soslow RA. Molecular insights into the classification of high-grade endometrial carcinoma. Pathology. 2018;50(2):151–161. 29246451 10.1016/j.pathol.2017.09.010

[24] Lu N, Liu J, Ji C, et al. MiRNA based tumor mutation burden diagnostic and prognostic prediction models for endometrial cancer. Bioengineered. 2021;12(1):3603–3620. 34252354 10.1080/21655979.2021.1947940 PMC8806700

[25] Dou Y, Kawaler EA, Cui Zhou D, et al. Proteogenomic Characterization of Endometrial Carcinoma. Cell. 2020;180(4):729–48 e26. 32059776 10.1016/j.cell.2020.01.026 PMC7233456

[26] Baretti M, Le DT. DNA mismatch repair in cancer. Pharmacol Ther. 2018;189:45–62. 29669262 10.1016/j.pharmthera.2018.04.004

[27] Liu J, Eckert MA, Harada BT, et al. m(6)A mRNA methylation regulates AKT activity to promote the proliferation and tumorigenicity of endometrial cancer. Nat Cell Biol. 2018;20(9):1074–1083. 30154548 10.1038/s41556-018-0174-4 PMC6245953

[28] Li Q, Wang C, Dong W, et al. WTAP facilitates progression of endometrial cancer via CAV-1/NF-kappaB axis. Cell Biol Int. 2021;45(6):1269–1277. 33559954 10.1002/cbin.11570

[29] Zhang Y, Yang Y. Effects of m6A RNA methylation regulators on endometrial cancer. J Clin Lab Anal. 2021;35(9):e23942. 34347888 10.1002/jcla.23942 PMC8418492

[30] Zou Z, Zhou S, Liang G, et al. The pan-cancer analysis of the two types of uterine cancer uncovered clinical and prognostic associations with m6A RNA methylation regulators. Mol Omics. 2021;17(3):438–453. 34110327 10.1039/d0mo00113a

[31] Xu X, Lai Y, Hua ZC. Apoptosis and apoptotic body: disease message and therapeutic target potentials. Biosci Rep. 2019;39(1). DOI:10.1042/BSR20180992 PMC6340950 30530866

